# Is salinity the main ecological factor that influences foliar nutrient resorption of desert plants in a hyper-arid environment?

**DOI:** 10.1186/s12870-020-02680-1

**Published:** 2020-10-07

**Authors:** Lilong Wang, Xinfang Zhang, Shijian Xu

**Affiliations:** 1 MOE Key Laboratory of Cell Activities and Stress Adaptations, School of Life Sciences, Lanzhou University, No. 222, Southern Tianshui Road, Lanzhou, 730000 China; 2 Naiman Desertification Research Station, Northwest Institute of Eco-Environment and Resources, Chinese Academy of Sciences, Lanzhou, 730000 China

**Keywords:** Nutrient retranslocation, Temperate desert, Leaf traits, Nutrient cycling, Sodium tress

## Abstract

**Background:**

Soil salinity is a major abiotic constraint to plant growth and development in the arid and semi-arid regions of the world. However, the influence of soil salinity on the process of nutrient resorption is not well known. We measured the pools of both mature and senesced leaf nitrogen (N), phosphorus (P), potassium (K), and sodium (Na) of desert plants from two types of habitats with contrasting degrees of soil salinity in a hyper-arid environment of northwest China.

**Results:**

N, P, K revealed strict resorption, whereas Na accumulated in senesced leaves. The resorption efficiencies of N, P, and K were positively correlated with each other but not with Na accumulation. The degree of leaf succulence drives both intra-and interspecific variation in leaf Na concentration rather than soil salinity. Both community- and species-level leaf nutrient resorption efficiencies (N, P, K) did not differ between the different habitats, suggesting that soil salinity played a weak role in influencing foliar nutrients resorption.

**Conclusions:**

Our results suggest that plants in hyper-arid saline environments exhibit strict salt ion regulation strategies to cope with drought and ion toxicity and meanwhile ensure the process of nutrient resorption is not affected by salinity.

## Background

Soils in desert environments are resource impoverished because the low and pulsed precipitation reduces soil nutrient availability by limiting the weathering of parent material and organic matter production and mineralization [1]. However, desert plants have adapted to these nutrient-poor habitats by employing a suite of leaf-level traits to conserve nutrients, including long tissue life span and tight nutrient recycling [2–4]. Nutrient resorption from senescing leaves is an important mechanism for plants to re-use mineral nutrition and makes them less dependent on external nutrient supply [5]. It has been estimated that, worldwide, on average, 60% of foliar nitrogen (N) and phosphorus (P) would be withdrawn into living tissues before leaf abscission [6]. Speculation about the importance of nutrient conservation has suggested that desert plants may rely more heavily on resorption than non-desert plants. However, there are controversial results regarding this hypothesis, with comparing data from seven desert shrubs to average values for non-desert shrubs, N and P resorption efficiency was higher in desert species (Killingbeck 1993), in contrast, six shrubs in Chihuahuan desert were no more efficient or proficient at resorbing N and P than non-desert shrubs (Killingbeck, 2001). The mixed results suggest that resorption may not be a simple function of soil fertility in desert ecosystems. Besides, the process of resorption may be influenced by abiotic factors, including drought and salinity, which commonly occur in arid environments [7].

Soil salinity is one of the most devastating environmental stresses, which causes osmotic and ionic stress to plants, and both will impose nutrient limitation on plant growth [1]. Generally, osmotic stress decreases the diffusion rate of nutrients in the soil to the absorbing root surface [8]. In contrast, ionic stress often causes unbalanced nutrient uptake because essential mineral nutrients such as NH_4_^+^ and K^+^ are replaced by Na^+^ in the rhizosphere zone [9]. Recent studies have shown that higher nutrient resorption efficiency is an adaptive strategy for several mangrove tree species to meet its nutrient requirement when facing salinity-induced nutrient limitation [10, 11]. In contrast to coastal salinization, soil salinity is a common phenomenon in arid environments, as desert soils are often saline due to the intense evaporation, especially within the inland river basin where the water table is relatively high [1, 12]. However, to date, few studies have examined this issue in arid environments, where plant nutrient resorption was often decreased by drought [13], thus, more attention should be paid to plants in arid saline environments.

Although N and P are crucial mineral nutrients for plant metabolism and functioning and limit plant growth worldwide [14], other elements, such as potassium (K) and sodium (Na), also have essential biochemical and physiological functions. For example, K plays a vital role in osmoregulation, respiration, photosynthesis, protein synthesis, and stomatal movement [15], while Na is an essential osmotic regulator for halophytic species and beneficial to many species at lower levels of supply [16]. However, it has been widely confirmed that both cellular and whole plant level nutrient homeostasis may be disrupted under Na stress [9]. Therefore, it is necessary to determine the relationship of resorption characteristics between Na and other mineral nutrients of plants in arid saline environments.

The Anxi Extra-arid Desert Reserve is located at the temperate desert in northwest China, Central Asia. Most areas of the reserve are occupied by gravel desert, where the soils are sandy with abundant gravels and extra-low moisture and salt content [17]. The gravel desert habitat (GDH) provides a proper habitat for extreme xerophytes. In contrast, since part of the reserve belongs to the Shule River basin (an interior drainage basin), salinization is a natural phenomenon in this area where the soils are less stony, higher in moisture content, and contain toxic levels of Na salts. A variety of halophytic desert plants inhabit the saline habitat (SH) [18]. Observational studies in such contrasting habitats provide a natural laboratory to examine the environmental constraints on nutrient resorption and give valuable information on the long-term adaptive response of plants to the hyper-arid saline environment. Hence, in this study, we wanted to test whether soil salinity is the main ecological factor that influences foliar nutrient resorption of desert plants in a hyper-arid environment. We compared both species- and community-level leaf elements resorption efficiencies in different habitats, and the effects of soil salinity and other soil properties on community-level nutrient resorption were quantified. Overall, we hypothesized plants found on saline soils would have lower green leaf nutrient concentration than those found on gravel desert due to the inhibition of nutrient uptake induced by ion toxicity and consequently be more dependent on nutrient resorption (i.e., have higher nutrient resorption efficiencies). Additionally, we hypothesized that among all soil properties, salinity is the driving factor affecting the characteristics of community-level nutrient resorption.

## Results

### Soil and vegetation characteristics

**Fig. 1:**
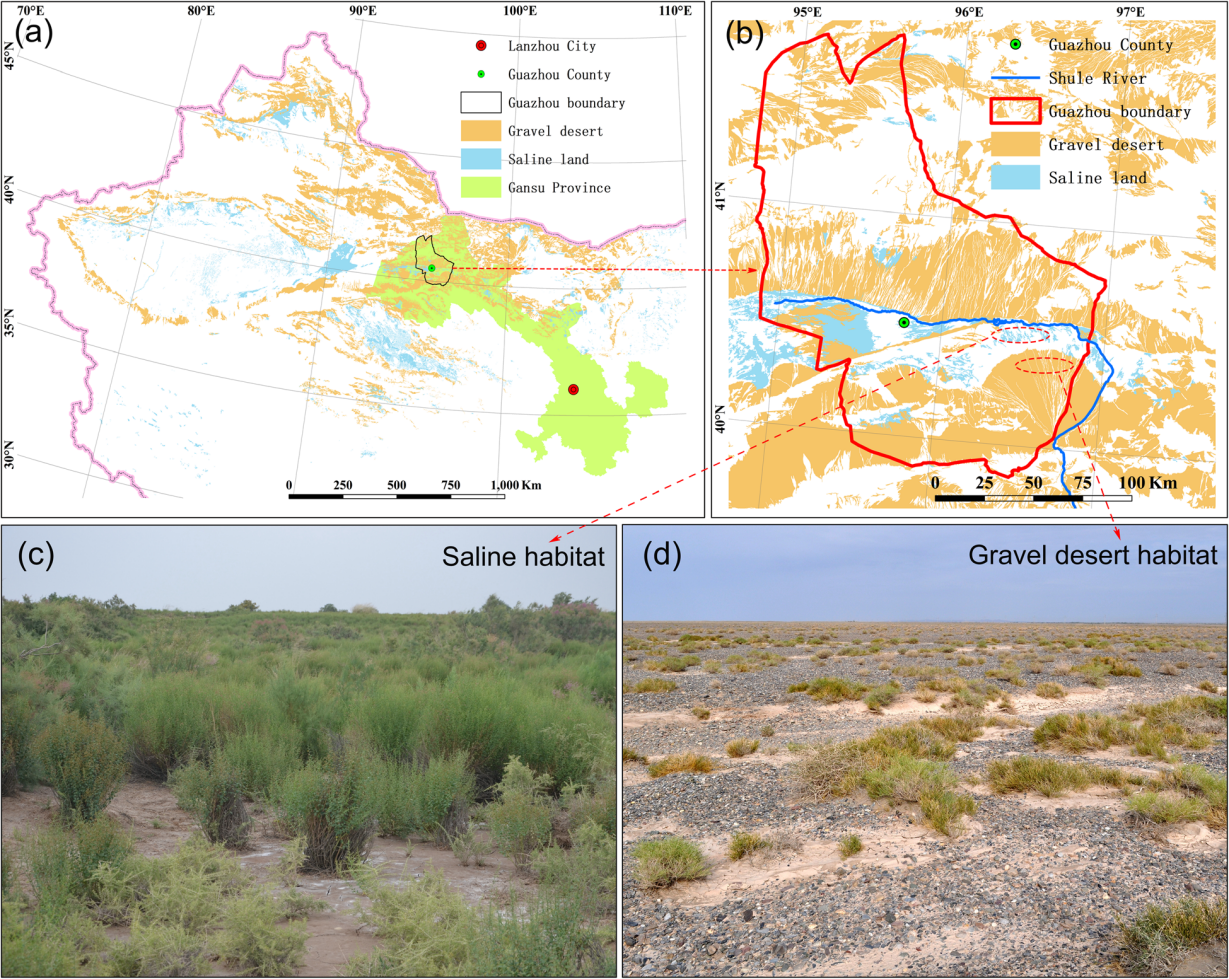
Distribution of gravel desert and saline land in northwest China (**a**) and in the study area (**b**), and examples of saline habitat (**c**) and gravel desert habitat (**d**) of the study area. The map depicted in (**a**) and (**b**) were plotted based on the 1:100000 desertification data of China, the data were free to acquire from the “Environmental & Ecological Science Data Center for West China, National Natural Science Foundation of China” (http://westdc.westgis.ac.cn). The photos depicted in (**c**) and (**d**) were taken by the author in July 2016

**Fig. 2:**
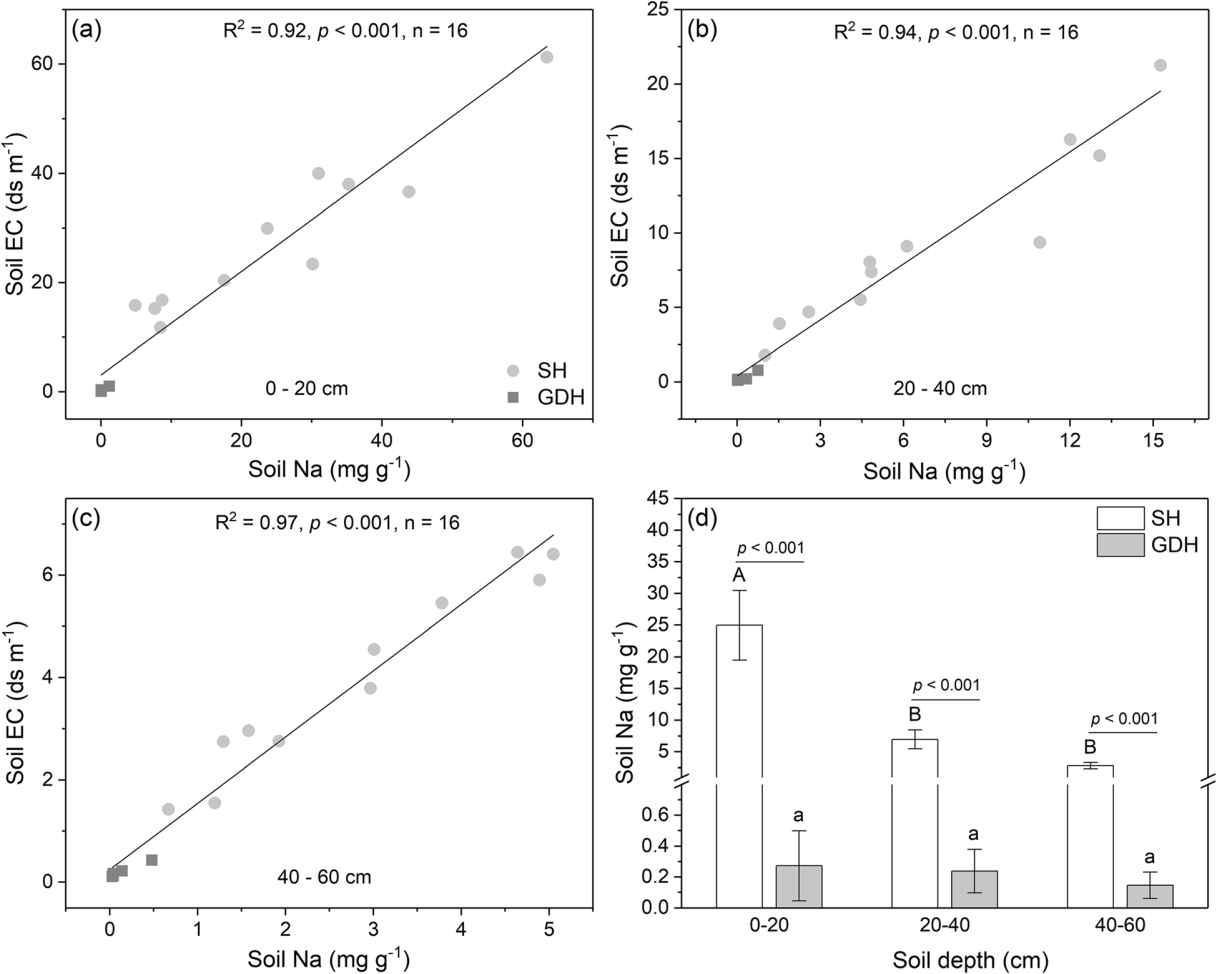
The relationship between soil soluble Na content and Soil electrical conductivity (EC) at different depth: **a** 0–20 cm; **b** 20–40 cm; **c** 40–60 cm. **d** The soil soluble Na content at different depths. Different uppercase and lowercase letters indicate significant differences (*p* < 0.05) at different soil depth in habitats (SH) and gravel desert habitats (GDH), respectively; the *P*-value above the bars indicates the difference between SH and GDH at the same soil depth. The correlations were evaluated by using standardized major axis regression

Considerable differences in vegetation characteristics were also observed (Fig. 1). The SH have significantly higher vegetation coverage, plant density, and species richness compared with GDH (Table 1). There were significant differences in soil properties between the two habitats. Soil pH, WC, and EC were significantly higher at the SH than the GDH (Table 1). A clear linear relationship between soil soluble Na content and Soil EC was observed (Fig. 2), indicating that soil Na content increases with increasing soil EC. Soil EC decreases significantly with increasing soil depth in SH, but the other soil properties did not vary among soil depth. Soil total N content was higher at the SH than the GDH, in contrast, there was no significant difference in soil total P and plant-available N and P content between the two types of habitats (Table 1).

**Table 1:** Soil properties and vegetation characteristics of the saline habitats (SH) and the gravel desert habitats (GDH) in the study area

	Properties	SH (*n* = 11)			GDH (*n* = 5)			Difference between SH and GDH		
		0–20 cm	20–40 cm	40–60 cm	0–20 cm	20–40 cm	40–60 cm	0–20 cm	20–40 cm	40–60 cm
Soil properties	pH	8.56 ± 0.05A	8.50 ± 0.06A	8.49 ± 0.06A	7.98 ± 0.06a	7.87 ± 0.03a	7.87 ± 0.04a	**	**	**
	WC (%)	15.3 ± 1.76A	18.5 ± 1.67A	16.6 ± 1.97A	2.31 ± 0.85a	2.49 ± 0.51a	2.87 ± 0.65a	**	**	**
	EC (ds m-1)	28.1 ± 4.12A	9.32 ± 1.65B	4.00 ± .052C	0.38 ± 0.17a	0.28 ± 0.13a	0.21 ± 0.06a	**	**	**
	TN (mg g^−1^)	0.74 ± 0.10A	0.68 ± 0.13A	0.53 ± 0.08A	0.25 ± 0.03a	0.22 ± 0.01a	0.22 ± 0.02a	**	*	*
	TP (mg g^−1^)	0.61 ± 0.05A	0.68 ± 0.05A	0.65 ± 0.04A	0.74 ± 0.04a	0.70 ± 0.06a	0.68 ± 0.05a	*NS.*	*NS.*	*NS.*
	AN (mg Kg^−1^)	12.1 ± 2.43A	10.3 ± 2.54A	14.8 ± 3.10A	17.3 ± 4.36a	15.4 ± 2.30a	13.2 ± 1.68a	*NS.*	*NS.*	*NS.*
	AP (mg Kg^−1^)	13.9 ± 3.84A	11.3 ± 2.33A	11.8 ± 3.50A	17.1 ± 3.18a	15.3 ± 5.16a	12.7 ± 2.73a	*NS.*	*NS.*	*NS.*
Vegetation characteristics	Coverage (%)	34.8 ± 4.67			13.9 ± 2.39			*		
	Density (individual m^−2^)	12.8 ± 4.14			0.61 ± 0.15			*		
	Richness (100 m^−2^)	7.63 ± 4.23			3.68 ± 0.45			*		

Different uppercase and lowercase letters indicate significant differences at *p* < 0.05 level (analyzed by one-way ANOVA) in soil properties at different depth in SH and GDH, respectively

*WC* soil water content, *EC* soil electric conductivity, *TN, TP* total soil nitrogen and phosphorus content, *AN, AP* plant-available nitrogen and phosphorus content

* and ** indicate significant differences at *p* < 0.05 and *p* < 0.01 level respectively (analyzed by independent sample *t*-test) in vegetation characteristics and soil properties at the same depth between SH and GDH respectively, *NS* represent non-significant difference

### Leaf chemistry and resorption efficiencies

**Fig. 3:**
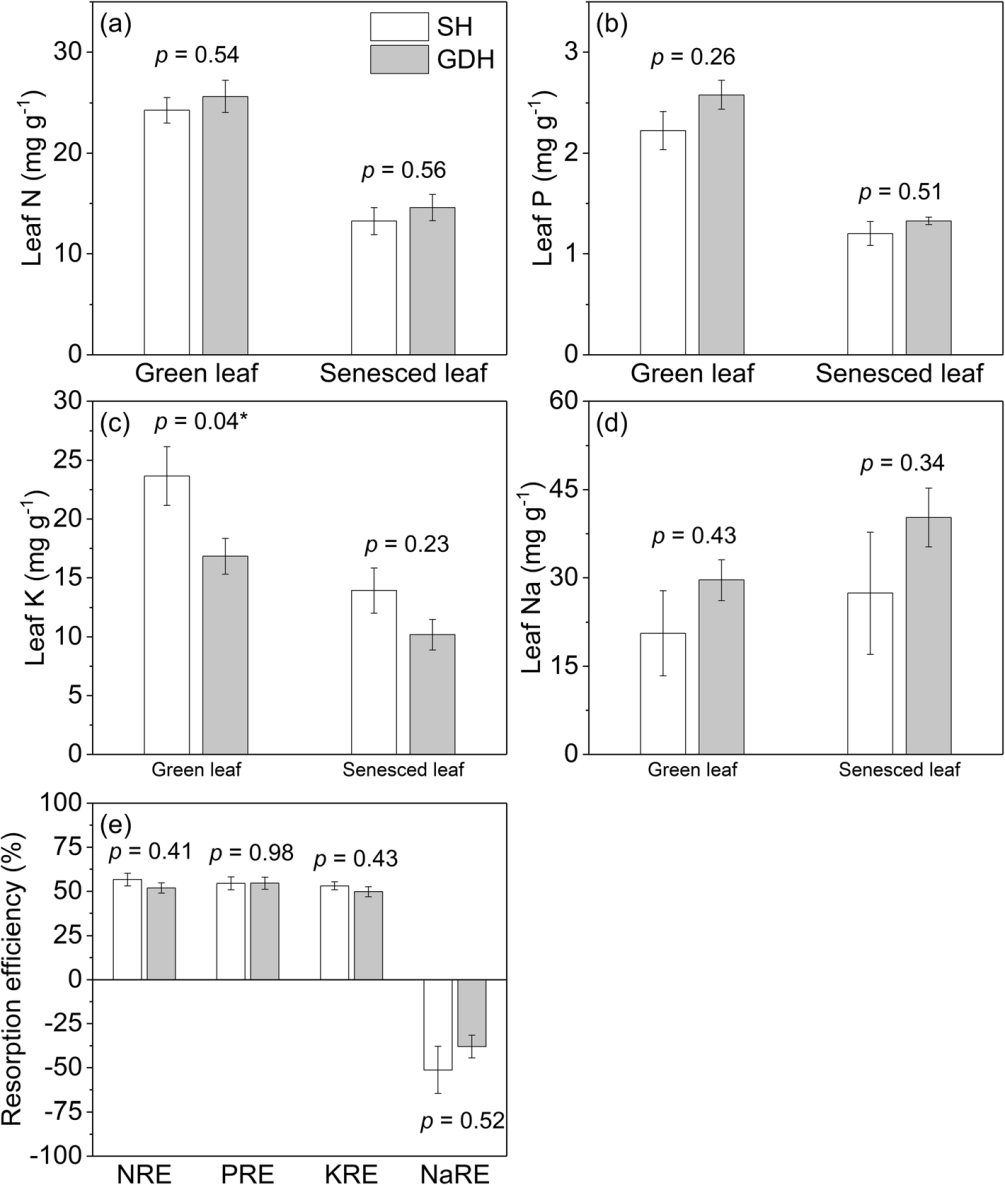
Community-level leaf N, P, K, Na concentration, and resorption efficiency in saline habitats (SH) and gravel desert habitats (GDH). The *P*-value (analyzed by independent sample *t*-test) above the bars indicates the difference of community-level traits in different habitats

**Fig. 4:**
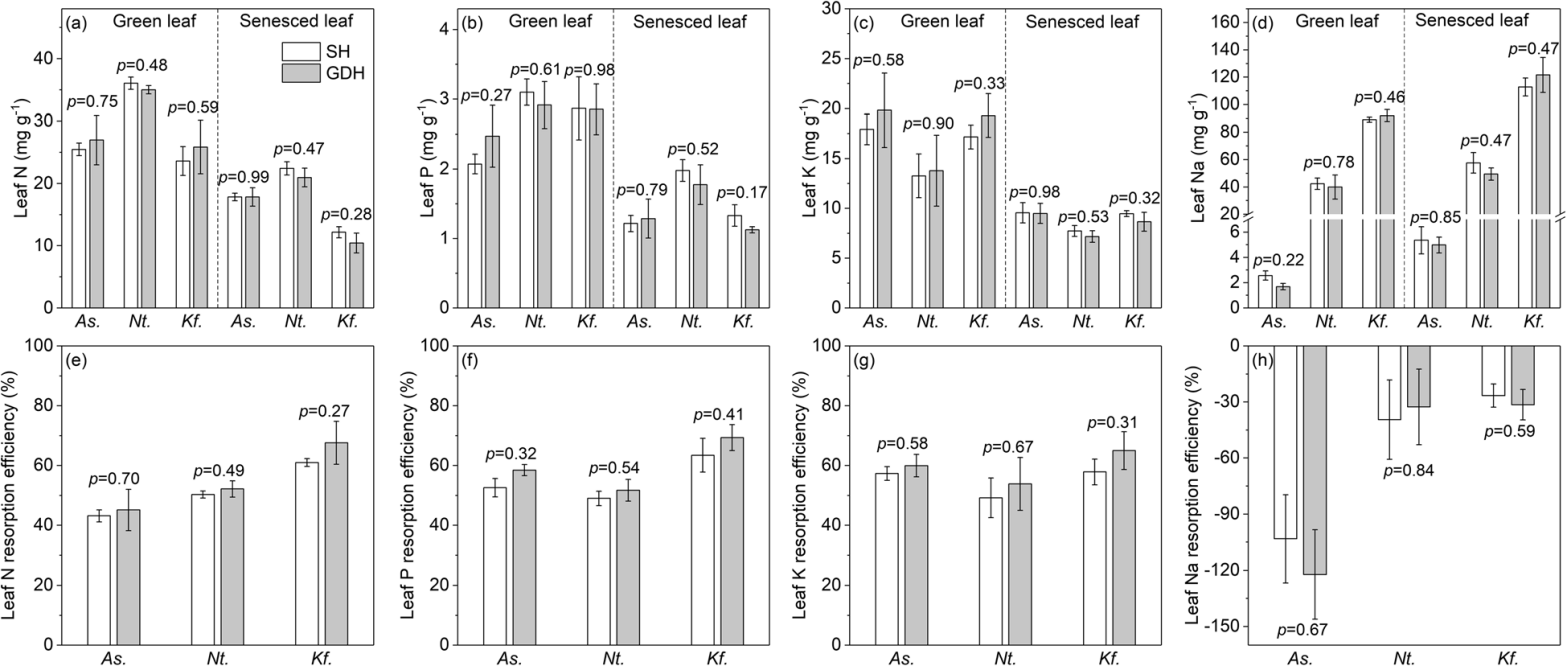
Leaf N, P, K, Na concentration, and resorption efficiency of the coexisting species in saline habitats (SH) and gravel desert habitats (GDH). *As*., *Alhagi sparsifolia*; *Nt*., *Nitraria tangutorum*; *Kf*., *Kalidium foliatum*. The *P*-value (analyzed by independent sample *t*-test) above the bars indicates the difference in traits of the coexisting species in different habitats

At the community level, there were no significant differences in both green and senesced leaf N, P, Na concentration between SH and GDH (all *P* > 0.26; Fig. 3a, b, d), but the green leaf K concentration was higher in SH than in GDH (*P* = 0.04; Fig. 3c), suggest that the dominant species in SH have higher green leaf K than that in GDH. At the species level, no significant differences were found both in green and senesced leaf N, P, K, Na concentrations of the three coexisting species between SH and GDH (all *P* > 0.17; Fig. 4a, b, c, d).

N, P, K showed resorption during leaf senescence; in contrast, Na tended to accumulate in senesced leaves. Across the study area, the community level NRE varied from 40.2 to 75.7% (mean 55.1%), PRE from 38.0 to 75.0% (mean 54.5%), KRE from 31.6 to 66.7% (mean 50.1%), NaRE from − 1.3 to − 150.5% (mean − 47.1%). No differences in community-level mean N, P, K, or Na resorption were found between SH and GDH (all *P* > 0.41; Fig. 3e). Similarly, the RE of N, P, K, Na concentrations in leaves of the three coexisting species also did not differ with habitat types (all *P* > 0.27; Fig. 4e, f, g, h).

### Effects of soil properties on resorption

**Fig. 5:**
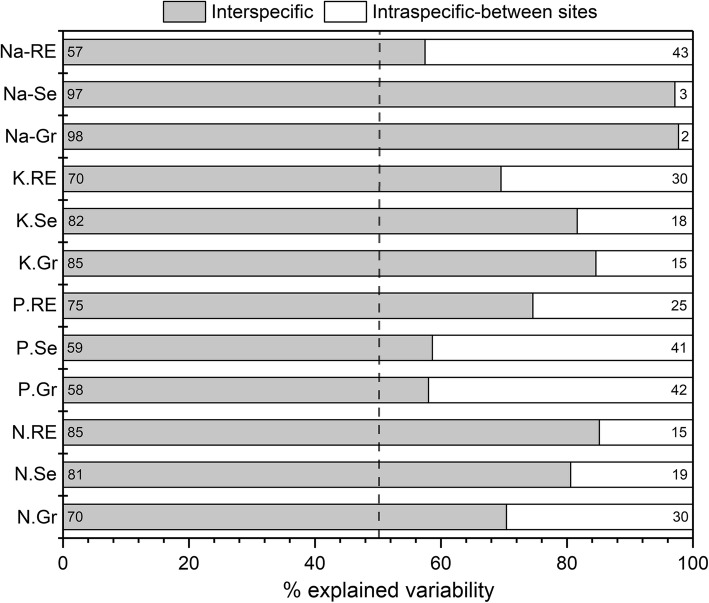
The relative contributions of interspecific and intraspecific between-site variability effects to the explained variation (analyzed by one-way ANOVA) for leaf elements concentrations and resorption efficiencies (RE)

At the community level, hierarchical partitioning analysis indicated that between 25% (in NRE) to 80% (in KRE) variation of leaf elements REs were accounted for by the soil properties, and the coefficients of determination (R^2^) increased with increasing soil depth (Table 2). NRE was not closely correlated with all soil properties at any depth. In contrast, PRE was significantly associated with AP (20–40 and 40–60 cm) (*P* < 0.05); KRE was significantly associated with TP (0–20 cm), TN (20–40 cm), pH and WC (40–60 cm) (*P* < 0.05); NaRE was significantly associated with TP (0–20 and 20–40 cm) (*P* < 0.05) (Table 2). Overall, at the community level, leaf elements REs were more closely related to soil fertility (i.e., TN, TP, AN, AP). Specifically, soil EC was not correlated with the REs of any elements (Table 2). Moreover, decomposition of the variation in leaf elements concentrations and REs showed that more than 50% of the total variation came from interspecific variability, indicating that the community-level leaf traits were mainly driven by species turnover rather than between sites intraspecific variability (Fig. 5).

**Table 2:** Fraction of variance (%) accounted for soil properties in community level element resorption efficiencies

Element	Soil depth (cm)	Full model (R^2^)	Soil properties						
			EC	pH	WC	TN	AN	TP	AP
NRE	0–20	0.25	13.65	5.47	16.12	8.25	14.07	17.25	25.19
PRE		0.29	10.65	6.73	38.52	14.67	2.08	11.81	15.53
KRE		0.56	4.03	13.60	19.00	19.17	10.54	28.56*	5.09
NaRE		0.46	13.54	15.97	5.00	15.16	2.59	41.46*	6.27
NRE	20–40	0.52	20.59	3.99	10.86	7.70	14.17	11.39	31.30
PRE		0.63	5.31	4.57	17.75	10.00	5.08	14.80	42.49*
KRE		0.73	1.53	20.91	23.28	25.33*	13.66	10.60	4.70
NaRE		0.66	4.02	3.65	9,81	4.44	15.90	43.03*	19.15
NRE	40–60	0.50	6.33	22.04	11.47	6.34	7.13	15.23	31.45
PRE		0.69	3.14	1.71	11.18	4.57	26.71	16.03	36.66*
KRE		0.80	12.23	25.87*	25.77*	18.62	1.13	8.56	7.83
NaRE		0.80	4.08	1.89	9.00	8.75	37.27*	21.10	17.90

*RE* indicates resorption efficiency. * indicates significance at *p* < 0.05 level (analyzed by Hierarchical Partitioning). *EC* electrical conductivity, *WC* water content, *TN, TP* total soil nitrogen and phosphorus content, *AN, AP* plant-available nitrogen, and phosphorus content

### Leaf trait correlations

At the species-level, there was a significant positive correlation between leaf N and P concentrations, regardless of whether the phylogenetic relatedness was removed (Table S1). At the community-level, PRE was positively correlated with NRE and negatively correlated with NaRE (all *P* < 0.01; Table 3). At the species level, significant correlations were only detected between NRE and PRE (*P* < 0.01). However, the correlations between NRE, PRE, and KRE became significant after the removal of phylogenetic relatedness (all *P* < 0.01; Table 3). There were significant positive correlations between LSI and leaf Na concentration both at community and species level (all *P* < 0.01, Table 4, Fig. S1). NRE was significantly positively correlated with LSI at the species level (*P* = 0.02). In contrast, the correlation became insignificant after the removal of phylogenetic relatedness (*P* = 0.82). No significant correlations were detected between LSI and RE of P, K, Na (all *P* > 0.05, Table 4).

**Table 3:** Covariations among element resorption efficiencies (RE)

Bivariate relationship	Community (*n* = 16)		Species (*n* = 21)		Species PIC (*n* = 20)	
	*r*	*p*	*r*	*p*	*r*	*p*
NRE - PRE	**0.63**	**< 0.01**	**0.83**	**< 0.01**	**0.90**	**< 0.01**
NRE - KRE	−0.28	0.29	0.10	0.66	**0.58**	**< 0.01**
PRE - KRE	−0.08	0.76	−.026	0.25	**0.71**	**< 0.01**
NRE - NaRE	−0.19	0.48	−0.17	0.46	0.16	0.49
PRE - NaRE	− **0.58**	**0.02**	− 0.41	0.06	−0.04	0.88
KRE - NaRE	− 0.02	0.95	−0.03	0.89	0.23	0.33

Significant relationships at *p* < 0.05 level are presented in bold (analyzed by Pearson correlation)

*RE* indicates resorption efficiency, *PIC* phylogenetically independent contrasts

**Table 4:** Covariations between leaf succulence index (LSI) and elements concentrations and resorption efficiencies

Bivariates	Community (*n* = 16)		Species (*n* = 21)		Species PIC (*n* = 20)	
	*r*	*p*	*r*	*p*	*r*	*p*
LSI - Ngr	0.17	0.53	0.11	0.64	−0.32	0.17
LSI - Nse	−0.01	0.96	−0.30	0.19	−0.30	0.20
LSI - NRE	0.07	0.80	**0.49**	**0.02**	−0.06	0.82
LSI - Pgr	0.43	0.10	0.17	0.45	−0.21	0.36
LSI - Pse	−0.05	0.86	−0.17	0.47	−0.23	0.34
LSI - PRE	0.38	0.15	0.43	0.05	−0.15	0.52
LSI - Kgr	−0.15	0.57	0.14	0.53	−0.09	0.69
LSI - Kse	−0.16	0.56	0.19	0.41	0.01	0.95
LSI - KRE	0.13	0.63	−0.10	0.68	−0.21	0.38
LSI - Nagr	**0.90**	**< 0.01**	**0.88**	**< 0.01**	**0.77**	**< 0.01**
LSI - Nase	**0.88**	**< 0.01**	**0.87**	**< 0.01**	**0.72**	**< 0.01**
LSI - NaRE	0.16	0.55	0.09	0.70	0.19	0.41

Significant relationships at *p* < 0.05 level are presented in bold (analyzed by Pearson correlation)

*LSI* indicates leaf succulence index, *gr* indicates green leaf, *se* indicates senesced leaf, *RE* indicates resorption efficiencies, *PIC* phylogenetically independent contrasts

## Discussion

### Leaf nutrient resorption does not differ between the two contrasting habitats

We hypothesized that plants in SH might rely more heavily on nutrient resorption than those in GDH, and consequently have higher NuRE. Because Na toxicity induced by salt stress may inhibit plant nutrient uptake. As reported by recent studies, several mangrove tree species can adapt to N limitation caused by salt stress by improving NRE [10, 11]. However, in contrast to our expectation, neither leaf chemistry nor NuRE differs significantly between the two contrasting habitats (Figs. 3, 4), suggesting that soil salinity played a weak role in influencing the process of nutrient resorption. We believe this may be partly explained by the mechanisms of salt tolerance, as if ion toxicity is avoided during salt stress, nutrients uptake and transportation would not be adversely affected, as many halophytes grow optimally in the presence of salt [19, 20].

To survive and reproduce in saline conditions, two main strategies are employed by the plants in the study area to deal with salt, i.e., compartmentation and exclusion. Among the three coexisting species, *Alhagi sparsifolia* employs salt-exclusion strategy [21] and has the lowest leaf Na concentration (2.35 mg g^− 1^ on average), even lower than the national averages (8.91 mg g^− 1^) of terrestrial plants in China [22]. In addition, Na concentrations in leaves of *A.sparsifolia* also did not differ between SH and GDH (Fig. 4), indicating that Na uptake was rejected at the root level in saline conditions. Because plants with salt exclusion strategy can prevent salt ions from entering the transpiration stream, thereby maintaining a favorable internal environment in leaf [23]. In contrast, plants with salt compartmentation strategies are often highly succulent and need to take up and sequester a substantial amount of Na in the vacuole as osmoticum [24], as we found in the present study that leaf Na concentration increases with leaf succulence index (Table 4, Fig. S1). There were also no significant differences in leaf Na concentrations of the two coexisting succulent species between SH and GDH (Fig. 4), demonstrating that Na accumulation also occurs actively in non-saline conditions. As indicated by previous research that Na concentrations in the leaves of succulent halophytes are strictly restricted and do not change with external salinity [19]. Interestingly, based on the field investigation, we found that five of the 11 SH and all five GDH were dominated by succulent species (LSI > 500), which implies that succulent plants are more adapted to hyper-arid environments. Because succulence can serve to improve energy returns on leaf investment by replacing expensive carbon structures with water and allowing for increased carbon investment in drought and salt tolerance [25]. Additionally, no significant correlations were detected between the concentrations of Na and N, P, and K (Table S1), suggesting that Na accumulation does not affect concentrations of the key nutrients in leaves of the species studied and protects them from ion toxicity. Thus, plants in the study area have evolved strict salt ion regulation mechanisms in coping with drought and salt stresses under long-term selective pressure.

Although the relationships between nutrient resorption and soil nutrients in the natural conditions are still being debated, a large number of fertilization experiments have indicated that NuRE decreased with increasing soil nutrient availability [26–28], which suggests that nutrient resorption is mainly affected by soil available nutrients rather than soil total nutrients. In the present study, the total soil N content in SH was significantly higher than in GDH, but the soil available N contents did not differ significantly between the two habitats because soil salinity may adversely affect the decomposition and mineralization rates of organic matter [29]. In contrast, soil P is mainly supplied by the weathering of parent material [30], neither total nor available P contents differ significantly between SH and GDH. Thus, there is no need for plants to rely more heavily on nutrient resorption in SH than those in GDH under similar nutrient supply conditions.

### Plant nutrient conservation in hyper-arid environments

As the three most important mineral nutrients for plant growth and development, N, P, and K are necessary for the metabolism of proteins, enzymes, and nucleic acids and are highly mobile in the phloem [31]. Our results indicated that N, P, and K showed strict resorption across the species studied (Table S2), which are generally in agreement with the findings of previous research [32, 33]. However, the mean values of NRE (varying from 26.46 to 83.02%, with a mean value of 50.91%), PRE (ranging from 32.35 to 75.63%, with a mean value of 53.46%, Table S2) and KRE (varying from 33.4 to 71.6%, with a mean value of 49.4%) were lower than the global average of 62.1, 64.9 and 70.1% [6]. The unexpected low NuRE may be attributed to the less proficient resorption. By introducing the concept of resorption proficiency, plants are highly proficient in nutrient resorption if they reduce the concentrations of N and P in senescing leaves to < 7 mg g^− 1^ and < 0.5 mg g^− 1^, respectively [5]. According to this criterion, none of the species studied were highly proficient in P resorption, and only one species was highly proficient in N resorption (Table S2). Moreover, we found that both species- (*r* = − 0.47, *P* < 0.05) and community-level (*r* = − 0.60, *P* < 0.05) NRE increased significantly with decreasing N concentration of senesced leaves, which suggests that the lower NuRE is mainly caused by the less proficient nutrient resorption. Similarly, in the semi-arid region of northern China, plants growing in N limited conditions were also less proficient in N resorption and showed lower NRE compared with global averages [34, 35]. The results seem to be unexpected because selection pressure in nutrient impoverished environments should make plants to reach complete resorption [2, 27]. However, on the other hand, these findings suggest that drought instead of soil salinity is the main limiting factor, which exerts a negative control on nutrient resorption of plants in hyper-arid environments [12, 13].

Studies have shown that interspecific N and P concentrations of green leaves are tightly correlated [36, 37]. This is because, from the perspective of physiology, leaf N and P are strongly inter-dependent in several plant metabolic processes [38]. However, the correlation between leaf N and P may be decoupled in the face of nutrient enrichment as a result of luxury consumption [39, 40]. Here, we observed that whether the phylogenetic affiliation is considered or not, the N and P concentrations of mature green leaves were significantly correlated. This correlation remains the same after the process of nutrient resorption (Table S1), suggesting that the concentrations of these two coupled nutrients in leaves of the species studied are not beyond its functional requirements. The relationship of KRE to the NuRE of other nutrients has not been reported to date. Interestingly, we found that the interspecific KRE was not correlated with NRE and PRE. However, these correlations became significant after the phylogenetic affiliations were removed, indicating that phylogeny may mask the relationship of KRE to NRE and PRE. Together, the results shown here provide evidence that, in the study area, resorption of the key nutrients is strongly linked under nutrient-limited conditions.

Since phloem transport is the only way to achieve leaf nutrient resorption in vascular plants, phloem mobility is an essential feature for those elements to be retranslocated from senescing leaves [41]. Similar to N, P, and K, Na is also highly mobile in the phloem [9]. However, in the present study, 18 of the 21 species studied showed significant accumulations of Na in senesced leaves, which agrees with the findings in non-desert plants [32, 42]. This is because the metabolic toxicity of Na is thought not to differ between halophytes and glycophytes, all (or at least most) Na taken up for osmotic adjustment has to be sequestered in vacuoles and kept away from sensitive metabolic pathways [20]. Thus, to maintain normal metabolism, resorption of Na from senescing leaves is prohibited, especially for those halophytic plants with abnormally high leaf Na concentrations. Overall, strict resorption of N, P, and K are keystone mechanisms to conserve nutrients, and Na accumulation is crucial for plants to avoid ion toxicity and cope with drought stress. These two mechanisms may not interfere with each other and jointly maintain the normal growth and reproduce of the plants in the hyper arid environments.

## Conclusions

Our study provides a test of the influence of soil salinity on nutrient resorption in a hyper-arid saline environment. We showed that N, P, and K revealed strict resorption, whereas Na accumulated in senesced leaves. The NRE, PRE, and KRE were positively correlated with each other across the species studied when the phylogenetic affiliations were removed. Both community- and species-level leaf NuRE (N, P, K) did not differ between the two contrasting habitats suggesting that soil salinity played a weak role in influencing nutrient resorption. The Na concentrations in leaves of the coexisting species were determined by specific Na regulation strategies rather than soil salinity. The accumulation of Na does not affect the resorption of N, P, and K. Overall, the results in the present study suggest that strict salt ion regulation strategies are vital for the plants in the study area to cope with drought and ion toxicity and meanwhile ensure that the process of nutrient resorption is not affected by soil salinity. Our findings on the patterns of elements resorption in leaves of desert plants may help understand plant adaption strategy and nutrient cycling processes in hyper-arid environments. Further studies will be needed to assess the potential resorption for the plants in the study area through repeated, annual sampling and recognize the environmental and biological driving factors over time.

## Methods

### Study area

The study area, the Anxi Extra-arid Desert National Reserve, is located between 39°52′-41°53′N, 94°45′-97°00′E, Gansu Province, northwest China. This area belongs to the intersection of Qinghai-Tibet Plateau and Mongolia-Xinjiang Desert. Since the warm and moist air from the Indian Ocean is obstructed by the Qinghai-Tibet Plateau, the climate in this region is temperate continental, with a mean annual temperature ranging from 7.6 °C–8.2 °C. The mean annual precipitation is only about 45 mm, but the mean annual evapotranspiration is over 3000 mm [43]. Thus, the aridity index (the ratio of mean annual precipitation to mean annual potential evapotranspiration) is below 0.02, representing a hyper-arid environment. The wind is an important erosive force in this region, and most areas of the Anxi Reserve are typical gravel desert with limited soil surface protection by the vegetative cover. The Shule River is the only perennial stream in the study area, which originates from the Qilian mountains and is recharged by meltwater. Eventually, it disappears after infiltration and evaporation in the piedmont alluvial plain [44]. The northernmost part of the Anxi Reserve is covered by the Shule River Basin, where soil salinity occurs naturally as a result of intense evaporation and shallow groundwater table. Soil salinity leads to significantly higher soil water content, electrical conductivity, and pH in the saline area than the gravel desert. Therefore, based on the presence of salinization, the Anxi Nature Reserve mainly consists of two contrasting habitats, i.e., gravel desert habitats (GDH) and saline habitats (SH) (Fig. 1).

### Sampling of leaf and soil

In July 2016, 16 sites were established in the study area where vegetation had been characterized previously [18], eleven of which were in SH, and five were in GDH. At each site, 20 × 20 m plots were set up based on the flat area to exclude variation in vegetation owing to changes in topography. The corners of each plot were marked with red wooden sticks. Because only one plot was selected at each site, we paid particular attention to selecting plots where the vegetation was visually most representative in terms of species abundance and composition. We considered this setup preferable to higher replication of random plot in desert environments. Within each plot, all individual plants were counted and identified to species. The percentage canopy cover of each species was also estimated, and the relative cover (%) of each species was then estimated as a fraction of the total canopy cover of each plot. During the peak growing period (middle of July), sun-exposed and fully expanded green leaves were collected from at least five individuals of each species (marked with red metal tag). Fully senesced leaves (often yellow) were collected from the tagged individuals at each plot by gently flicking the branch or leaf. The sampled leaves were rinsed with deionized water to remove surface salts and dust by using a spray bottle in situ. For each species, 20–60 g leaves (mixed uniformly with the individual) were collected, of which about 10 g were stored in the icebox to keep fresh, and the rest were stored in paper envelopes for chemical analysis. Overall, 150 leaf samples of 21 species were collected across the study area (Table S3), and three of them coexist in two habitats. The species identification is based on the taxonomic classification of *Flora in Desertis Reipublicae Populorum Sinarum* [45] and *Halophytes in China* [46]. The formal identification of the samples is undertaken by the corresponding author, who is a professor of botany at the College of Life Sciences, Lanzhou University, and the specimen information of the species involved in this study are available at “China Plant Species Information System” (http://www.iplant.cn). Field sampling was authorized and assisted by the Anxi Extra-arid Desert Reserve. We confirmed that the field studies did not involve endangered or protected plant species and declare that the work reported here complies with the current laws of China and the IUCN Policy Statement on Research Involving Species at Risk of Extinction.

Triplicate soil samples of three depth (0–20 cm, 20–40 cm, 40–60 cm) were randomly taken by an auger (in SH) or a shovel (in GDH) within each plot, where each replicate was composed by a mixture of three adjacent soil cores. The soil samples were divided into three parts. Part one was stored in an aluminum box and weighted in situ and then dried at 105 °C for 24 h in the laboratory to determine the water content. Part two was packed in a paper envelope and then air-dried for chemical analysis. Part three was packed in a polyethylene bag and stored in an icebox to keep fresh for the analysis of soil available N content.

### Chemical analysis and leaf trait measurement

All samples collected were taken to the laboratory, where different analyses were performed. Leaf samples stored in paper envelopes were oven-dried at 70 °C to a constant weight and ground into a fine powder using a ball mill (MM200, Retsch, Haan, Germany) to enable chemical analysis. The total N content in soil samples (air-dried, 0.15 mm sieved) and in leaf samples were measured by an elemental analyzer (FLASHEA 1112 Series CNS Analyzer, Thermo, USA), and the total P content was measured using the ammonium molybdate method after persulfate oxidation [18]. Leaf K and Na and soil soluble Na concentration were analyzed by the atomic absorption spectroscopy (iCETM 3300, Thermo, USA). Before analysis, leaf samples (fine powder) were digested in the mixed acid (HNO_3_:HClO_4_:H_2_SO_4_ = 8:1:1, volume ratio) at 420 °C, and soil samples (2 mm sieved, air-dried) were extracted by deionized water (soil: water = 1:10). The soil pH and electrical conductivity (EC) were measured on 1:2.5 and 1:5 soil: water extracts, respectively. Soil samples were extracted with KCl (2 M L^− 1^), and the soil extracts were used to determine the soil available N content (ammonium and nitrate) with a continuous flow spectrophotometer (FIAstar5000, FOSS, Denmark). Soil available P content was determined by the molybdenum blue-ascorbic acid method after extracting the soil sample with 0.5 M L^− 1^ NaHCO_3_ [47].

Fresh leaf samples stored in the icebox were transported to the laboratory where the fresh mass (M_f_) were determined immediately. The area of fresh leaf samples (A_f_) was measured by a photographic method [48] and analyzed using ImageJ (National Institutes of Health, USA, http://imagej.nih.gov/ij). The samples were oven-dried at 70 °C to a constant weight (M_d_). Leaf succulence index (LSI) was calculated as g water m^− 2^ ([M_f_ - Md] / A_f_) [42].

### Calculation of nutrient resorption characteristics

Community-level means for leaf element concentrations and resorption efficiencies were calculated by using species relative cover as a weighting factor [49, 50]. Leaf element resorption efficiencies (RE) were quantified as the proportional withdrawal of a nutrient or element during senescence and expressed as:

$$ RE=\left(1-\frac{Nu_{senesced}}{Nu_{green}}\times MLCF\right)\times 100\% $$

Where Nu_green_ and Nu_senesced_ are mass-based element concentrations in green and senesced leaves. Considering the mass loss during the process of leaf senescence, the mass loss correction factor (MLCF) could be used to compensate for the underestimation of RE [51]. In this study, the MLCF was calculated as the ratio of the dry mass of senesced leaves and the dry mass of green leaves [6].

### Statistical analysis

Data were tested for normality using the Kolmogorov-Smirnov test and for the equality of error variance using Levene’s test. “Independent sample *t*-test” was used to test the differences in each leaf trait between SH and GDH at the species level (means of coexisting species in two habitats) and community level (cover weighted means) separately. “One-way ANOVA” was performed separately to disentangle the relative contributions of interspecific and intraspecific (between sites) variability effects to the explained variability for each trait. “Hierarchical Partitioning (HP)” analysis was used to examine the effects of soil factors on community-level leaf nutrient resorption efficiency. “Standard major axis regression (SMA)” was used to determine the relationship between leaf Na concentration and leaf succulence index. “Pearson correlation” was used to determine the relationship between the resorption efficiency of different leaf elements. In addition, pairwise correlations among different traits were re-evaluated by calculating “Phylogenetically independent contrasts (PIC)” to remove the phylogenetic relatedness among species due to shared evolutionary history [52]. The phylogenetic tree of the species was constructed using the online tool Phylomatic (http://www.phylodiversity.net/phylomatic/phylomatic.html) based on Angiosperm Phylogeny Group III (APG III) classification [53]. All the analyses were carried out with R 3.6.1. (R Development Core Team, available from www.r-project.org/), of which the “Independent sample *t*-test,” “One-way ANOVA” and “Pearson correlation” was performed through the “stats” package; the “HP” analysis was performed through the “hier.part” package; the “SMA” was performed through “smatr” package; the “PIC” was performed through “ape” and “picante” package.

## Supplementary information

**Additional file 1: Table S1.** Covariations among leaf elements contents. **Table S2** Mean values of leaf elements contents and resorption efficiencies of the species across the study area. **Table S3** Specimen information corresponding to the sampled species in this study. **Fig. S1** The relationship between leaf succulence index (LSI) and green leaf Na contents at (a) community level and (b) species level. (c) phylogenetic independent contrast correlations at the species level. SH, saline habitats; GDH, gravel desert habitats. SH-species, species in saline habitats, GDH species, species in gravel desert habitats; Co-species, coexisting species in saline habitats, and gravel desert habitats. The correlations were calculated using standardized major axis regression (SMA).

## Data Availability

All data generated or analyzed during this study are included in this published article and its supplementary information files.

## Abbreviations

N: Nitrogen
P: Phosphorus
K: Potassium
Na: Sodium
NuRE: Nutrient resorption efficiency (such as NRE, PRE KRE, NaRE)
SH: Saline habitats
GDH: Gravel desert habitats
LSI: Leaf succulence index
