# Supplementary material for: Is salinity the main ecological factor that influences foliar nutrient resorption of desert plants in a hyper-arid environment?
Source: BMC Plant Biol. 2020 Oct 7;20:461. doi: 10.1186/s12870-020-02680-1 (PMC7539515; doi:10.1186/s12870-020-02680-1)
Supplement: Supplementary file 1 — Additional file 1: Table S1. Covariations among leaf elements contents. Table S2 Mean values of leaf elements contents and resorption efficiencies of the species across the study area. Table S3 Specimen information corresponding to the sampled species in this study. Fig. S1 The relationship between leaf succulence index (LSI) and green leaf Na contents at (a) community level and (b) species level. (c) phylogenetic independent contrast correlations at the species level. SH, saline habitats; GDH, gravel desert habitats. SH-species, species in saline habitats, GDH species, species in gravel desert habitats; Co-species, coexisting species in saline habitats, and gravel desert habitats. The correlations were calculated using standardized major axis regression (SMA). [file 12870_2020_2680_MOESM1_ESM.pdf]

Is salinity the main ecological factor that influences foliar nutrient resorption of desert plants in a hyper-arid environment?

Lilong Wang[1,2], Xinfang Zhang[1], Shijian Xu[1*]

[1]MOE Key Laboratory of Cell Activities and Stress Adaptations, School of Life Sciences, Lanzhou University, Lanzhou 730000, China.
[2]Naiman Desertification Research Station, Northwest Institute of Eco-Environment and Resources, Chinese Academy of Sciences, Lanzhou 730000, China.

[*]Corresponding author: Shijian Xu
Address: No. 222, Southern Tianshui Road, Lanzhou 730000, China.
E-mail address: xushijian@lzu.edu.cn

Table S1 Covariations among leaf elements contents.

	Ngr	Nse	Pgr	Pse	Kgr	Kse	Nagr	Nase
Ngr		0.73^{***}	0.70^{***}	0.55^{**}	0.19	0.12	0.15	0.25
Nse	0.75^{**}		0.47^{*}	0.72^{***}	-0.15	-0.24	-0.30	-0.24
Pgr	0.65^{**}	0.49^{*}		0.77^{***}	0.35	0.28	0.31	0.40
Pse	0.37	0.54^{*}	0.84^{**}		0.161	0.06	-0.10	-0.07
Kgr	0.21	0.16	0.44	0.46^{*}		0.96^{***}	0.05	0.11
Kse	0.12	0.01	0.15	0.06	0.87^{***}		0.08	0.13
Nagr	-0.07	-0.28	0.20	0.04	-0.18	-0.19		0.99^{***}
Nase	0.07	-0.20	0.32	0.06	-0.11	-0.11	0.97^{***}	

The gr and se represent green and senesced leaf respectively. *, ** and *** represent significant correlations at $P < 0.05$, $P < 0.01$, $P < 0.001$ level respectively. The right part is interspecific correlation, and the left part is phylogenetically independent contrast (PIC) correlation.

Table S2 Mean values of leaf elements contents and resorption efficiencies of the species across the study area.

Species	Ngr (mg g^{-1})	Nse (mg g^{-1})	NRE (%)	Pgr (mg g^{-1})	Pse (mg g^{-1})	PRE (%)	Kgr (mg g^{-1})	Kse (mg g^{-1})	KRE (%)	Nagr (mg g^{-1})	Nase (mg g^{-1})	KRE (%)
Achnatherum splendens	19.34	14.12	34.52	1.38	0.99	35.50	17.54	10.85	44.51	2.09	2.32	-11.13
Agropyron cristatum	19.87	15.19	49.72	2.76	2.01	52.32	24.62	10.68	71.58	3.40	2.69	20.84
Ajania fruticulosa	38.97	29.59	44.29	5.94	3.03	62.52	30.30	17.26	58.22	4.60	13.06	-184.0
Alhagi sparsifolia	25.85	17.83	43.62	2.17	1.23	54.03	18.40	9.53	57.98	2.35	5.26	-107.98
Apocynum venetum	18.86	3.53	83.02	2.30	0.83	67.87	41.47	29.83	35.06	7.30	11.13	-81.63
Asterothamnus centraliasiaticus	23.87	17.34	38.07	3.39	2.14	46.09	31.30	20.17	45.24	3.23	4.17	-31.81
Ephedra przewalskii	15.68	11.73	33.56	1.80	1.12	44.42	8.22	5.45	40.86	0.75	0.49	32.86
Glycyrrhiza uralensis	25.12	16.19	43.88	2.14	1.18	53.07	12.04	6.65	51.86	0.78	1.21	-56.83
Gymnocarpos przewalskii	19.21	15.80	26.46	1.46	1.10	32.35	10.26	7.04	38.60	2.56	3.58	-40.13
Halostachys caspica	27.88	10.18	67.65	4.56	1.48	71.20	27.34	20.25	34.36	83.62	128.05	-53.13
Kalidium foliatum	24.44	11.52	63.46	2.86	1.25	65.64	17.97	9.17	60.52	90.21	116.13	-28.37
Karelinia caspia	15.60	9.15	49.15	2.76	1.63	48.84	4.29	2.62	47.06	38.22	41.81	-9.40
Lycium ruthenicum	26.49	12.62	59.75	2.05	1.36	43.92	35.44	20.30	52.00	25.44	32.53	-29.77
Nitraria sphaerocarpa	40.93	23.72	58.20	4.25	1.78	69.32	20.27	11.57	59.18	35.36	62.08	-76.12
Nitraria tangutorum	35.66	21.88	50.96	3.03	1.90	50.01	13.45	7.52	50.93	41.42	54.50	-36.89
Phragmites australis	23.76	16.57	42.56	1.92	1.42	38.91	14.71	7.57	56.58	1.93	1.62	15.33
Reaumuria songarica	19.91	12.65	44.45	1.83	1.16	43.64	5.49	3.86	37.95	34.75	39.74	-14.48
Salsola arbuscula	27.35	12.28	61.56	2.46	1.14	59.83	34.51	26.72	33.40	33.32	46.06	-38.41
Salsola passerina	17.25	9.67	59.46	1.67	0.86	63.16	23.69	14.01	57.78	40.32	56.35	-40.19
Sophora alopecuroides	30.12	21.22	40.62	2.01	1.31	45.18	5.00	3.64	38.66	4.66	9.14	-96.13
Sympegma regelii	32.66	11.73	73.61	3.57	1.18	75.63	19.89	9.24	66.43	39.69	61.00	-54.41

The gr and se indicates green and senesced leaf respectively; RE indicates resorption efficiency.

Table S3 Specimen information corresponding to the sampled species in this study.

Species	Specimen Number	Specimen Photo
Achnatherum splendens	HNWP 166886	http://v5.cvh.ac.cn/spm/HNWP/167114
Agropyron cristatum	HNWP 167086	http://v5.cvh.ac.cn/spm/HNWP/167086
Ajania fruticulosa	HNWP 1501702	http://v5.cvh.ac.cn/spm/HNWP/1501702
Alhagi sparsifolia	HIMC 0038441	http://v5.cvh.ac.cn/spm/HIMC/0038441
Apocynum venetum	HIMC 0026402	http://v5.cvh.ac.cn/spm/HIMC/0026402
Asterothamnus centraliasiaticus	HNWP 00015045	http://v5.cvh.ac.cn/spm/HNWP/HNWP00015045
Ephedra przewalskii	HNWP 0233812	http://v5.cvh.ac.cn/spm/HNWP/0233812
Glycyrrhiza uralensis	HNWP 196571	http://v5.cvh.ac.cn/spm/HNWP/196571
Gymnocarpos przewalskii	HNWP 150707	http://v5.cvh.ac.cn/spm/HNWP/150707
Halostachys caspica	PE 01196825	http://v5.cvh.ac.cn/spm/PE/01196825
Kalidium foliatum	PE 00541739	http://v5.cvh.ac.cn/spm/PE/00541739
Karelinia caspia	PE 02037422	http://v5.cvh.ac.cn/spm/PE/02037422
Lycium ruthenicum	PE 01197565	http://v5.cvh.ac.cn/spm/PE/01197565
Nitraria sphaerocarpa	PE 01197178	http://v5.cvh.ac.cn/spm/PE/01197178
Nitraria tangutorum	PE 01197185	http://v5.cvh.ac.cn/spm/PE/01197185
Phragmites australis	HNWP 168182	http://v5.cvh.ac.cn/spm/HNWP/168182
Reaumuria songarica	HNWP 0234439	http://v5.cvh.ac.cn/spm/HNWP/0234439
Salsola arbuscula	HIMC 0038401	http://v5.cvh.ac.cn/spm/HIMC/0038401
Salsola passerina	HIMC 0037677	http://v5.cvh.ac.cn/spm/HIMC/0037677
Sophora alopecuroides	HNWP 166693	http://v5.cvh.ac.cn/spm/HNWP/166693
Sympegma regelii	HNWP 0234302	http://v5.cvh.ac.cn/spm/HNWP/0234302

The details and photos of the specimens corresponding to the species in this study are available at "China Plant Species Information System" (http://www.iplant.cn).

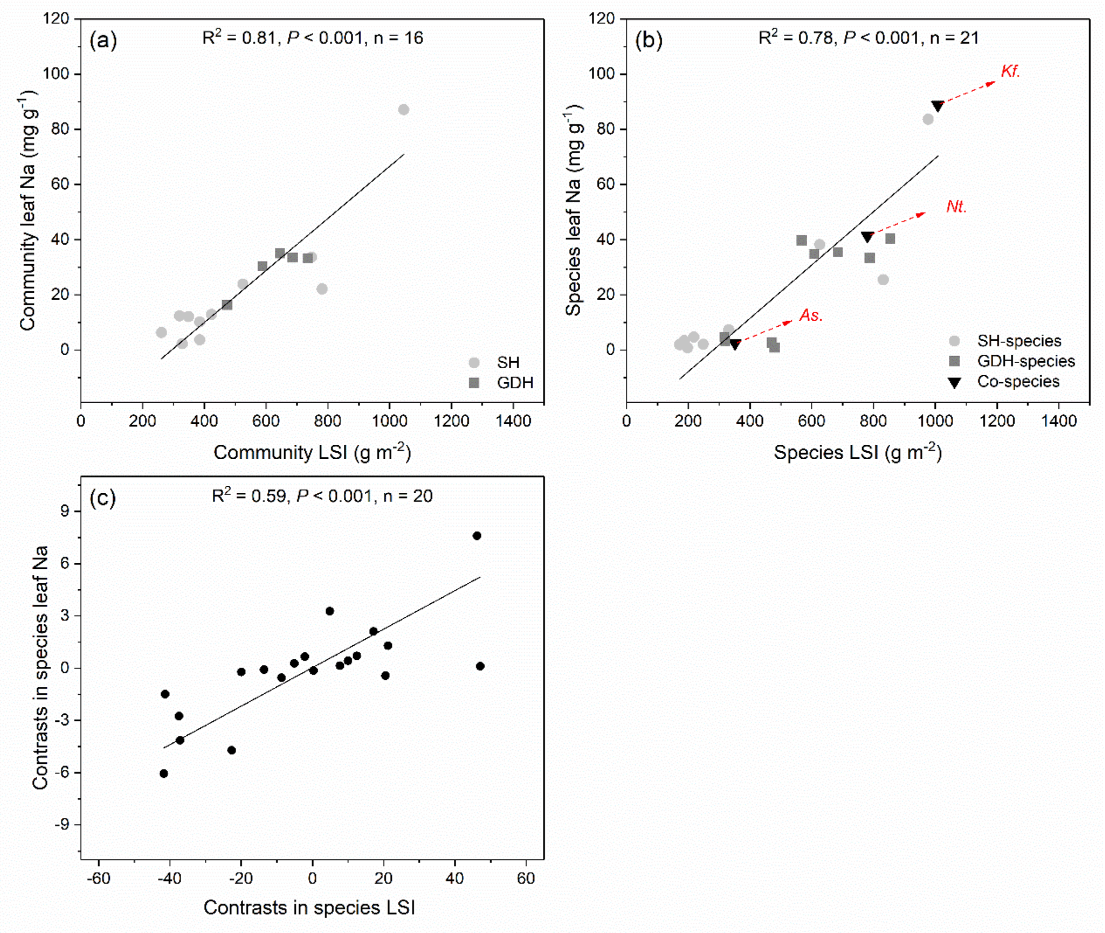

Fig. S1 The relationship between leaf succulence index (LSI) and green leaf Na contents at (a) community level and (b) species level. (c) phylogenetic independent contrast correlations at species level. SH, saline habitats; GDH, gravel desert habitats. SH-species, species in saline habitats, GDH species, species in gravel desert habitats; Co-species, coexisting species in saline habitats and gravel desert habitats. The correlations were calculated using standardized major axis regression (SMA).
